# College Life Adjustment, Life Stress, Career Barriers, and Career Decision-Making Self-Efficacy of College Students Who Have Experienced COVID-19 in South Korea

**DOI:** 10.3390/healthcare10040705

**Published:** 2022-04-11

**Authors:** Jeong-Hye Park, Se-Won Kang

**Affiliations:** 1 Department of Nursing, Gyeongsang National University, Dongjin-ro 33, Jinju-si 52725, Gyeongsangnam-do, Korea; masternur@gnu.ac.kr; 2 Department of Nursing, Dongseo University, 47 Jurye-ro, Sasang-gu, Busan 47011, Korea

**Keywords:** adaptation to college life, life stress, career barriers, career decision-making self-efficacy

## Abstract

University life is challenging for students, given the college life adjustment, life stress, career barriers, and career decision-making self-efficacy required. COVID-19 has made this even more complex. This study investigated the relationships among these factors in college students who experienced COVID-19. Data were collected using an online cross-sectional structured survey of 1242 university students from December 2020 to January 2021. Data were analyzed via independent-sample *t*-tests, one-way ANOVAs, Pearson’s correlation coefficient, multiple regression, and logistic regression, using SPSS. College life adjustment was significantly correlated with life stress (*r* = −0.255, *p* < 0.001), career barriers (*r* = −0.429, *p* < 0.001), and career decision-making self-efficacy (*r* = 0.531, *p* < 0.001). The regression analysis showed that career barriers, career decision-making self-efficacy, and grade level had a total explanatory power of 33.7% for college life adaptation. The logistic regression analysis showed that the odds ratio (OR) of career barriers being low in the group with a high college life adaptation score was 2.045 (*p* < 0.001), and the OR of career decision-making self-efficacy being high was 4.107 (*p* < 0.001), as compared to the group with a low score. To increase college students’ adaptation to college life, career support programs that improve career barriers and career decision-making self-efficacy should be developed.

## 1. Introduction

Adaptation to college life plays an important role in preparing college students to enter society and forms a framework for them to become adults [1]. In college, there are developmental tasks, during which students must establish their identity, adapt to a new environment, and consider and decide on their career path [2]. In Korea, these tasks, which are usually completed during adolescence, are often postponed to the university period, due to the heterogeneous and passive lifestyle many individuals adopt during middle and high school. This time is primarily spent concentrating on university entrance exams [3]. Therefore, Korean college students often complain about identity confusion and maladaptation to college life. This occurs as they engage in important developmental tasks during middle and high school, and adapt to the new university environment [4]. In addition, college students experience psychological confusion while adapting to various social and cultural living environments, as well as tension and frustration in college life. They must solve almost all of these challenges autonomously [3].

College students experience many life stressors in college life. These include managing credits, time, and personal relationships, and being concerned about their careers and employment [5]. They experience stress in interpersonal relationships, such as those with friends, family, and professors, as well as challenges in academic, economic, future, and value issues [6]. If this stress is not properly managed, individuals experience maladaptive symptoms that may adversely impact their quality of life, happiness, and life satisfaction [7].

In addition, career barriers are college students’ main concern. Career barriers are factors that hinder career planning and the realization of career goals and factors that make career decisions difficult [8]. Many college students complain about adjusting to college life because of career barriers related to their career decisions [9]. The internal and external difficulties perceived by college students who have vocational skills and have the task of deciding on a career may affect their adaptation to college life [10]. Career barriers include objective external factors that prevent an individual from advancing in society and internal conflicts or problems recognized by the individual. Lent, Brown, and Hackett (1994) [11] explained career barriers using social cognitive career theory. Social cognitive career theory emphasizes the cognitive and personal variables that can affect an individual’s career development, as well as external and contextual factors that restrict or support the individual. Individual factors influence contextual factors, and contextual factors can mediate the process of converting an individual’s career interest into career goals and career performance.

However, when there are factors that hinder one’s career path, if the obstacles can be overcome, their negative impact may be reduced. Self-efficacy is an individual’s belief in their ability to manage and cope with difficult situations [12], and it is applicable to career considerations. Career decision-making self-efficacy refers to the degree of self-confidence one feels regarding successfully accomplishing tasks related to career decisions [13]. This variable may affect college students’ career development [14].

Personal factors, contextual/environmental factors, and experiential factors influence self-efficacy, and self-efficacy can mediate the interrelationships among career interest, career goals, and career choice behaviors. In addition, a stronger perception of career barriers is linked to lower confidence in performing tasks related to career decision making; this decreased confidence may lead to avoidance of the tasks related to career decision making [15].

Adaptation to college life is a multidimensional phenomenon. Whether the individual adapts to a given situation may depend on whether that person appropriately responds to various needs [16]. Adaptation to college life may be the result of interactions with elements of the individual’s environment. These include interpersonal, emotional, stress, and academic aspects [17,18]. University life in the COVID-19 context has significantly changed perceptions of university life compared to pre-COVID-19. Before COVID-19, college students’ daily lives involved freely belonging to various groups and participating in activities and relationships. This occurred while allocating appropriate time for classes, activities, and leisure around their schools. However, during the COVID-19 pandemic, college students’ adaptation to college life also changed.

Although various studies have been conducted on college life adaptation, no study has comprehensively examined college students’ life stress, career barriers, and career decision-making self-efficacy. A large-scale survey of college students’ results has not yet been reported. No studies to date have analyzed these factors, which are related to college life adaptation among college students who have experienced COVID-19.

### Study Purpose

This study examined the relationships among life stress, career barriers, and career decision-making self-efficacy to identify the factors that affect adaptation to college life among college students who have experienced COVID-19. The specific study objectives were as follows:

(1) To investigate college students’ degree of college life adaptation, life stress, career barriers, and career decision-making self-efficacy.
(2) To investigate the relationships among college life adaptation, life stress, career barriers, and career decision-making self-efficacy.
(3) To investigate the factors that influence their college life adjustment.

## 2. Materials and Methods

### 2.1. Study Design

This was a quantitative and cross-sectional study conducted online. It was a descriptive and inferential study investigating the effects of life stress, career barriers, and career decision-making self-efficacy on adaptation to college life of college students who have experienced COVID-19.

### 2.2. Study Participants

The study participants were 1242 out of 4504 students attending a local university in South Korea, who were recruited via convenience sampling and completed the survey. These participants experienced the COVID-19 situation for approximately 1 year, i.e., two semesters: the first and second semesters of 2020.

The average age of the participants was 23.1 years. Of these persons, 644 students (51.9%) were male and 598 students (48.1%) were female. Students were almost equally distributed across university years, with 285 students (22.9%) in first year, 303 students (24.4%) in second year, 312 students (25.1%) in third year, and 335 students (27.5%) in fourth year. Most of the participants belonged to the life science college (365 students; 29.4%). Of the participants, 878 (70.7%) were living at home (See Table 1).

### 2.3. Data Collection

Data were collected online using structured questionnaires for 8 days, from 29 December 2020 to 5 January 2021. Data collection was conducted at the Student Counseling Center of ‘K’ University in Korea. An online survey was conducted among all students enrolled at the university. The survey procedures complied with the Personal Information Protection Act and the university’s personal information processing policy. When the Student Counseling Center notified the student body that data collection was taking place, through the website and department, students could voluntarily access the online survey link during the survey period. After reading the participation agreement, if they agreed to participate, they could complete the agreement and thereafter the survey. The researcher requested the use of data according to university procedures and received approval. When universities provide data to researchers, the personal information of participants is deleted in accordance with the Personal Information Protection Act and the institution’s privacy policy (http://www.gntech.ac.kr/web/www/212 accessed on 7 April 2022). The questionnaire was sent to a total of 4504 people. Of the 1434 questionnaires returned, 1242 were included in the data analysis, after excluding 192 that contained insincere responses.

### 2.4. Study Instruments

#### 2.4.1. General Characteristics of Participants

The participants’ age, gender, grade, affiliated college, and residence type were investigated.

#### 2.4.2. College Life Adaptation

College life adaptation was measured using Jeong and Park’s College Life Adaptation tool [19]. This scale consists of 19 questions across five factors: interpersonal relationships, academic activities, career preparation, personal psychology, and social experiences. Each item is rated on a five-point Likert scale ranging from one point for “not at all” to five points for “strongly agree”. A higher score indicates a better adjustment to college life. The scale’s Cronbach’s alpha reliability coefficient at the time of development was 0.86. Jung’s [20] study reported a Cronbach’s alpha value of 0.88; in this study, the value was 0.90.

#### 2.4.3. Life Stress

Life stress was measured using the Life Stress Scale for college students by Chon, Kim, and Lee [21]. It consists of a total of 50 items, with four factors that pertain to interpersonal relationship problems: relationships with friends, relationships with the opposite gender, relationships with family members, and relationships with professors. Each item is rated on a five-point Likert scale ranging from one point for “not at all” to five points for “strongly agree”. A higher score indicates a greater life stress level. In other words, a higher score has a more negative meaning. In a previous study by Lee and Park [22], Cronbach’s alpha was 0.88; in this study, the value was 0.96.

#### 2.4.4. Career Barriers

Career barriers were measured using the Korean Career Indecision Inventory developed by Tak and Lee [23]. This tool consists of 22 items, with five factors: lack of career information, lack of self-identity, indecisiveness, lack of necessity recognition, and external barriers. Each item is rated on a five-point Likert scale ranging from one point for “not at all” to five points for “strongly agree”. A higher score indicates higher barriers (indecision and difficulty in career decision-making). In other words, a higher score has a more negative meaning. In a previous study by Jung [24], the reliability of the tool was 0.89. In this study, the reliability of the scale was 0.93.

#### 2.4.5. Career Decision-Making Self-Efficacy

Career decision-making self-efficacy was measured using the short form of the Career Decision-Making Self-Efficacy Scale of Betz, Klein, and Taylor [25]. This tool consists of five factors, with a total of 25 items: self-appraisal, occupation information, goal selection, planning, and problem-solving. Each item is rated on a five-point Likert scale ranging from one point for “not at all” to 5 points for “strongly agree”. A higher score indicates a higher self-efficacy in career decision-making. The Cronbach’s alpha reliability value of the tool at the time of development was 0.94. Cho’s scale [3] exhibited a reliability of 0.89. In this study, the reliability of the scale was 0.95.

### 2.5. Ethical Considerations

This study was conducted after review by the Ethics Committee of the Public Institutions of the Ministry of Health and Welfare of South Korea (IRB No. P01-202103-23-002). University students voluntarily accessed the online survey link during the survey period, read and filled out the consent form, and completed the survey. The ethical principles of anonymity, confidentiality, and the ability to withdraw from the study at any time were maintained. Informed consent letters were provided, and the participants were advised of what was required of them and the ethical principles governing their participation.

### 2.6. Data Analysis

The collected data were analyzed using IBM’s Statistical Package for the Social Sciences (SPSS), version 22.0 (IBM, Armonk, NY, USA). The analysis method was as follows:

1. Descriptive statistics (frequencies, percentages, mean scores, and standard deviations) were obtained for the scores on college life adjustment, life stress, career barriers, and career decision-making self-efficacy. Independent-sample *t*-tests and one-way ANOVA were used to assess the differences in the scores of each variable according to the participants’ gender and grade.
2. Pearson’s correlation coefficients were used to examine the relationships among college life adaptation, life stress, career barriers, and career decision-making self-efficacy.
3. Multiple regression analysis and logistic regression were used to identify factors associated with college life adaptation.
4. The significance level in all statistical tests was set at *p* < 0.05.

## 3. Results

### 3.1. Participants’ College Life Adjustment, Life Stress, Career Barriers, and Career Decision-Making Self-Efficacy

Participants’ mean score was 3.4 for college life adaptation, 2.3 for life stress, 2.6 for career barriers, and 3.4 for career decision-making self-efficacy on a five-point scale. There were no differences in the mean scores for each variable according to gender, affiliated college, or residence type. However, there were differences by grade level in college life adaptation (F = 10.84, *p* < 0.001), life stress (F = 5.14, *p* = 0.002), and career decision-making self-efficacy (F = 3.61, *p*= 0.013). These differences are presented in Table 2. Specifically, the college life adaptation score was higher in the third and fourth grades than the first and second grades. In addition, the life stress score was the lowest in the first year. The career decision-making self-efficacy score was higher in the first grade than in other grades.

### 3.2. Relationships among College Life Adaptation, Life Stress, Career Barriers, and Career Decision-Making Self-Efficacy

Table 3 shows the relationships among the participants’ college life adaptation, life stress, career barriers, and career decision-making self-efficacy. College life adjustment was significantly correlated with life stress (*r* = −0.255, *p* < 0.001), career barriers (*r* = −0.429, *p* < 0.001), and career decision-making self-efficacy (*r* = 0.531, *p* < 0.001). In addition, life stress and career barriers (*r* = 0.614, *p* < 0.001), life stress and career decision-making self-efficacy (*r* = −0.327, *p* < 0.001), and career barriers and career decision-making self-efficacy (*r* = −0.556, *p* < 0.001) showed statistically significant correlations. In other words, college life adjustment was positively related to career decision-making self-efficacy and negatively related to life stress and career barriers.

### 3.3. Factors Affecting College Life Adjustment

Regression analysis was performed to identify the factors associated with participants’ adaptation to college life. Grade, for which significant differences were found in the univariate analyses, was input as an independent variable after conversion to a dummy variable. The Durbin–Watson statistic was 1.934, which was close to the reference value of two; therefore, there was no autocorrelation. The tolerance value was greater than 0.1, i.e., between 0.47 and 0.97. The variance inflation factor (VIF) ranged from 1.03 to 2.11, i.e., below the reference value of 10. There was no multicollinearity between the independent variables.

The regression model for the factors affecting college life adjustment was statistically significant (F = 158.568, *p* < 0.001), with an explanatory power of 33.7%. Significant influencing factors were identified in the order of career barriers (β = −0.164, *p* < 0.001), career decision-making self-efficacy (β = 15.755, *p* < 0.001), and grade (β = 0.179, *p* < 0.001; see Table 4).

To understand the difference in college life adaptation according to detailed influencing factors, the odds ratio (OR) between the variable groups for each college life adaptation score group was calculated. On the basis of the mean, two groups (50% high-score group and 50% low-score group) were created. A base group and a comparison group were defined. In addition, the analysis adjusted for grade, for which variable differences existed in the univariate analysis. As a result, the OR of career barriers being low in the group with a high college life adaptation score was 2.045 (CI, 1.533–2.782, *p* < 0.001), and the OR of career decision-making self-efficacy being high was 4.107 (CI, 3.128–5.392, *p* < 0.001; see Table 5).

## 4. Discussion

This study investigated the factors associated with college life adaptation using a sample of university students from a region in Korea. The relationships among life stress, career barriers, and career decision-making self-efficacy and their impact on college life adjustment were analyzed.

### 4.1. Participants’ College Life Adjustment, Life Stress, Career Barriers, and Career Decision-Making Self-Efficacy

The average scores were 3.4, 2.3, 2.6 and 3.4 for college life adaptation, life stress, career barriers, and career decision-making self-efficacy, respectively. The college life adaptation score was higher than the value of 3.14 reported by Lee and Song [26] using the same tool, while Park and Kim [27] reported a similar value of 3.37.

It is conceivable that results may vary depending on the number of participants and sample group characteristics, even for previous studies that used the same scale. However, in each case, the score was above the scale midpoint, which represents positive college life adjustment.

The life stress score was 2.5, similar to that measured by Lee [28] for 348 college students. Despite the COVID-19 context, there were no significant differences in the scores. It is estimated that the students are adjusting well to the changed environment after experiencing this pandemic for 1 year.

The career barrier score was higher than the 2.04 reported by Seo and Lee [29] for college students who had not experienced COVID-19. There is a limit to comparing career barriers with only average scores because external barriers are found in the external environment and internal barriers form the psychological status of an individual. A comparative analysis of these factors is required in future studies.

In a study by Park and Kim [27], the score for career decision-making self-efficacy was also 3.4 for college students who collected data in 2018. This value is higher than the scale midpoint, which indicates that the degree of confidence in the career area was high. A score of 3.4 during the COVID-19 situation reflects both the university’s efforts to address pandemic-related restrictions via remote learning and the individual’s efforts to adapt to the environment. High self-efficacy in career decision-making may reduce the negative effects of career barriers and may be expected to act as a mediating variable in college life adaptation.

There were no gender-related differences in the scores of the variables. However, there were differences across grades in college life adaptation, life stress, and career decision-making self-efficacy. In particular, the college life adjustment score was higher in students in the upper grades than in the lower grades. However, the life stress score was also higher among the former group. The lower grades had higher self-efficacy scores for career decision-making. Therefore, it is necessary to develop a plan to increase college life adaptation for the lower grades, reduce life stress for the upper grades, and increase self-efficacy in career decision-making.

### 4.2. The Relationships among College Life Adaptation, Life Stress, Career Barriers, and Career Decision-Making Self-Efficacy

College life adjustment was significantly correlated with life stress, career barriers, and career decision-making self-efficacy. A lower life stress was correlated with fewer career barriers, and a higher self-efficacy in career decision-making was correlated with higher college life adjustment.

College life adjustment and life stress exhibited a significant negative correlation, which is consistent with the negative correlation (*r* = −0.570) in Kim and Son’s [30] study. However, the correlation in the current study was rather weak. College life adjustment and career barriers also exhibited a significantly negative correlation. This was generally consistently observed in previous studies.

There was a significant positive correlation between college life adaptation and career decision-making self-efficacy. This is similar to the results of Valenti and Faraci [16], who targeted Italian students. The positive expectations of opportunities for self-growth and the various experiences that college students have while attending college are related to career decision-making self-efficacy, which has the potential to affect their adjustment to college life.

### 4.3. Factors Associated with College Life Adjustment

The multiple regression analysis indicated that career barriers, career decision-making self-efficacy, and grade were associated with college life adjustment; the model explained 33.7% of the variance in college life adjustment. These results are congruent with those of previous studies [3,4,31], which reported that career-related variables appear to have a close relationship with college life adjustment. Many college students who visit college counseling centers complain about career-related problems, due to difficulties adjusting to college life. In addition, a lower grade revealed more cases of problems with college life adjustment.

These results were confirmed more specifically in the logistic regression analysis, which generated the ORs of each variable group for college life adaptation. The group with high college life adaptation scores was 2.045 times more likely to have low career barrier scores, and 4.107 times more likely to have high career decision-making self-efficacy.

To increase college life adaptation, good results may be obtained if career barriers are reduced and career decision-making self-efficacy is improved. Accordingly, it is necessary for universities to strengthen their career development programs, so that students may engage various coping skills when following their career path. It is necessary to seek ways to enhance career-related psychological reinforcement. Since there are many factors that hinder students’ adaptation to college life, it is reasonable to develop the individual’s own ability to appropriately respond, rather than to focus on temporarily removing external factors that promote maladjustment.

Adjustment to college life can be understood as adaptation to school situations, and it is important as a steppingstone to become a successful member of society [16,18]. The positive events experienced in the university environment serve as the foundation for one’s growth, and smoothly overcoming negative events is an important developmental task for an individual. In other words, when college students adapt well to school life, it means that they can fulfill their developmental tasks [3]. Park and Kim [27] reported that, similar to the results of this study, a higher college life adaptation is linked to higher self-efficacy in career decision making. This is because positive expectations for opportunities for self-growth and various experiences that college students can obtain while attending college may affect their adjustment to college life.

After the outbreak of COVID-19, college students’ daily lives experienced changes, such as the need to adapt to remote classes, failure of employment preparation plans, and a reduction in personal relationships. However, through a 1 year course, students are in the process of transforming their values, finding goals, transforming resources, growing, and becoming more independent [32,33]. This study’s results indicate that college students are still thinking about their career paths and are concerned about their careers, which affects their adaptation to university life. It is necessary for various career development programs to be developed and made accessible to students.

### 4.4. Study Limitations

As this study was conducted using convenience sampling, there were limitations with respect to generalizing the study’s results to all students. In addition, to analyze the directionality of relationships among variables, it would be necessary to trace the causal paths of the variables through structural model analysis in future studies.

The investigation was conducted 1 year after the outbreak of COVID-19; thus, there may have been changes in the study participants’ adaptation to university life as they progressed toward the early and late stages of the outbreak. This limitation cannot be strictly classified or controlled.

In addition, this study was a cross-sectional study; thus, an accurate comparison of the changes in college life adaptation of participants before and after the outbreak of COVID-19 was limited. In the future, it would be useful to test the variables by changing the study design.

In order to overcome the limitations of the research design, it is possible to propose experimental studies focused on developing a career decision self-efficacy program to improve college life adaptation and evaluate the effectiveness of the program.

## 5. Conclusions

This study is valuable because it provides basic data that may enhance students’ understanding of college adaptation by examining the relationships among life stress, career barriers, and self-efficacy in career decisions as variables that influence college life adjustment.

Through this study, career barrier, career decision self-efficacy, and grade were identified as major variables for college life adaptation. Career decisions and career decision-making self-efficacy were identified as factors that could affect college life adjustment and could be affected by grade level. In particular, career decision-making self-efficacy was found to be a major factor influencing college life adjustment.

In order to adapt to university life and various situations in modern society such as COVID-19, it will be necessary for each university to develop motivational and career employment programs geared toward developing self-efficacy, according to the characteristics of each grade level.

## Figures and Tables

**Table 1:** Participant characteristics (*N* = 1242).

Variables		*n* (%)	Mean (SD)	Min–Max
Age (Years)			23.1 (2.75)	19–60
Gender	Male	644 (51.9)		
	Female	598 (48.1)		
Grade	First	285 (22.9)		
	Second	303 (24.4)		
	Third	312 (25.1)		
	Fourth	335 (27.5)		
Affiliated college	Life science	365 (29.4)		
	Construction and environmental engineering	216 (17.4)		
	Convergence technology	302 (24.3)		
	Humanities and social sciences	141 (11.4)		
	Commerce	218 (17.6)		
Residence type	Home	878 (70.7)		
	Excluding home (e.g., dormitory)	364 (29.3)		

Note. SD: standard deviation.

**Table 2:** Scores of the variables and differences in characteristics by gender and grade.

Variables		Adjustment to College Life	Life Stress	Career Barriers	Career Decision-Making Self-Efficacy
		Mean (SD), Min 1–Max 5			
Total		3.4 (0.64)	2.3 (0.67)	2.6 (0.77)	3.4 (0.69)
Gender	Male	3.3 (0.67)	2.2 (0.70)	2.5 (0.81)	3.4 (0.72)
	Female	3.4 (0.61)	2.3 (0.64)	2.6 (0.71)	3.4 (0.64)
t(p)		−0.44 (0.661)	−1.13 (0.257)	−1.72 (0.086)	−0.49 (0.625)
Grade	First ^a^	3.2 (0.61)	2.1 (0.69)	2.6 (0.75)	3.5 (0.66)
	Second ^b^	3.3 (0.61)	2.3 (0.65)	2.6 (0.75)	3.4 (0.67)
	Third ^c^	3.4 (0.65)	2.3 (0.62)	2.6 (0.79)	3.3 (0.72)
	Fourth ^d^	3.5 (0.66)	2.3 (0.71)	2.6 (0.77)	3.4 (0.68)
F(p)		10.84 (<0.001) a,b < c,d	5.14 (0.002) a < b,c,d	0.57 (0.634)	3.61 (0.013) a > b,c,d
Affiliated college	Life science	3.4 (0.63)	2.2 (0.68)	2.6 (0.81)	3.4 (0.68)
	Construction and environmental engineering	3.4 (0.64)	2.3 (0.68)	2.5 (0.72)	3.4 (0.67)
	Convergence technology	3.3 (0.60)	2.2 (0.64)	2.6 (0.75)	3.3 (0.68)
	Humanities and social sciences	3.3 (0.91)	2.2 (0.67)	2.6 (0.74)	3.4 (0.67)
	Commerce	3.4 (0.64)	2.3 (0.70)	2.6 (0.77)	3.4 (0.72)
F(p)		1.12 (0.349)	1.28 (0.269)	0.40 (0.851)	1.83(0.105)
Residence type	Home	3.3 (0.63)	2.2 (0.67)	2.6 (0.76)	3.4 (0.66)
	Excluding home (e.g., dormitory)	3.3 (0.66)	2.2 (0.68)	2.5 (0.78)	3.4 (0.73)
t(p)		0.56 (0.610)	0.49 (0.625)	0.46 (0.648)	0.58 (0.560)

Note. ^a^: First Grade; ^b^: Second Grade; ^c^: Third Grade; ^d^: Fourth Grade; SD: standard deviation.

**Table 3:** Correlations among variables.

	Adjustment to College Life	Life Stress	Career Barriers
Adjustment to college life	1		
Life stress	−0.255 (*p* < 0.001)	1	
Career barriers	−0.429 (*p* < 0.001)	0.614 (*p* < 0.001)	1
Career decision-making self-efficacy	0.531 (*p* < 0.001)	−0.327 (*p* < 0.001)	−0.556 (*p* < 0.001)

**Table 4:** Factors associated with adjustment to college life (*N* = 1242).

Variables	B	SE	β	t	*p*
Life stress	−0.026	0.028	−0.028	−0.939	0.348
Career barriers	−0.137	0.028	−0.164	−4.872	<0.001
Career decision-making self-efficacy	0.411	0.026	0.440	15.755	<0.001
Grade	0.102	0.013	0.179	7.640	<0.001
*R*^2^ = 0.339, adjusted *R*^2^ = 0.337, F = 158.568, *p* < 0.001					

**Table 5:** Logistic regression of variables according to adjustment to college life ^a^.

Variables		B	*p*	OR	95% CI
Life stress					
Comparison group	Low-score group (*n* = 621, 50%)			1.182	
Base group	High-score group (*n* = 621, 50%)	0.167	0.232	1	0.898–1.554
Career barrier					
Comparison group	Low-score group (*n* = 623, 50.2%)			2.045	
Base group	High-score group (*n* = 619, 49.8%)	0.716	<0.001	1	1.533–2.728
Career decision-making self-efficacy					
Comparison group	High-score group (*n* = 603, 48.6%)			4.107	
Base group	Low-score group (*n* = 639, 51.4%)	0.413	<0.001	1	3.128–5.392

^a^ Adjustment to college life: (1) base group, low-score group—622 (50.1%), (2) comparison group, high score-group—620 (49.9%). Adjusted for grade. Abbreviations: CI, confidence interval; OR, odds ratio.

## Data Availability

Data sharing is not applicable.

## References

[1] Cousins C., Servaty-Seib H.L., Lockman J. (2017). College student adjustment and coping. Omega.

[2] Baker R.W., Siryk B. (1984). Measuring adjustment to college. J. Couns. Psych..

[3] Cho H.J. (2017). Analysis of the structural relationship among self-leadership, college adjustment, career decision-making self-efficacy. J. Career Educ. Res..

[4] Kim M.S. (2017). The influence of self-leadership and critical thinking disposition on college adaptation among nursing students. J. Korean Acad. Soc. Nurs. Edu..

[5] Graves B.S., Hall M.E., Dias-Karch C., Haischer M.H., Apter C. (2021). Gender differences in perceived stress and coping among college students. PLoS ONE.

[6] Arias-de la Torre J., Fernández-Villa T., Molina A.J., Amezcua-Prieto C., Mateos R., Cancela J.M., Delgado-Rodríguez M., Ortíz-Moncada R., Alguacil J., Redondo S. (2019). Psychological distress, family support and employment status in first-year university students in Spain. Int. J. Environ. Res. Public Health.

[7] Long R., Halvorson M., Lengua L.J. (2021). A mindfulness-based promotive coping program improves well-being in college undergraduates. Anxiety Stress Coping.

[8] Hashish E.A.A. (2019). The effect of career awareness on perceived career and talent development self-efficacy and career barriers among nursing students. J. Res. Nurs..

[9] Avery M., Westwood G., Richardson A. (2022). Enablers and barriers to progressing a clinical academic career in nursing, midwifery and allied health professions: A cross-sectional survey. J. Clin. Nurs..

[10] Steeb D.R., Zeeman J.M., Bush A.A., Dascanio S.A., Persky A.M. (2021). Exploring career development through a student-directed practicum to provide individualized learning experiences. Curr. Pharm. Teach. Learn..

[11] Lent R.W., Brown S.D., Hackett G. (1994). Toward a unifying social cognitive theory of career and academic interest, choice, and performance. J. Voca. Behav..

[12] Bandura A. (1977). Self-efficacy: Toward a unifying theory of behavioral change. Psychol. Rev..

[13] Taylor K.M., Betz N.E. (1983). Applications of self-efficacy theory to the understanding and treatment of career indecision. J. Vocat. Behav..

[14] Koçak O., Ak N., Erdem S.S., Sinan M., Younis M.Z., Erdoğan A. (2021). The role of family influence and academic satisfaction on career decision-making self-efficacy and happiness. Int. J. Environ. Res. Public Health.

[15] Lent R.W., Brown S.D., Hackett G. (2000). Contextual supports and barriers to career choice: A social cognitive analysis. J. Counsel. Psycho..

[16] Valenti G.D., Faraci P. (2021). Predicting university adjustment from coping-styles, self-esteem, self-efficacy, and personality: Findings from a survey in a sample of Italian students. Eur. J. Investig. Health Psychol Educ..

[17] Krajniak M.I., Pievsky M., Eisen A.R., McGrath R.E. (2018). The relationship between personality disorder traits, emotional intelligence, and college adjustment. J. Clin. Psychol..

[18] Bishop D.I., Hansen A.M., Keil A.J., Phoenix I.V. (2019). Parental attachment and adjustment to college: The mediating role of avoidant coping. J. Genet. Psychol..

[19] Jeong E.I., Park Y.H. (2009). Development and validation of the College Adjustment Scale. Korean J. Educ. Metho. Stud..

[20] Jung S.Y. (2019). A comparative study of college adjustment and life stress of nursing students by grades. J. Indus. Conver..

[21] Chon K.K., Kim K.H., Yi J.S. (2000). Development of the revised life stress scale for college students. Kor. J. Psychol. Health.

[22] Lee E.H., Park S.J. (2012). Validity and application of the Life Stress Scale for University Students. J. Educ. Res..

[23] Tak J., Lee K.H. (2003). Development of the Korean Career Indecision Inventory. J. Career Assess..

[24] Jung C.S. (2021). The effect of adult attachment of university students on their career barriers: Focused on the mediating effects of resilience. J. Acad. Ind. Technol..

[25] Betz N.E., Klein K.L., Taylor K.M. (1996). Evaluation of a short form of the Career Decision-making Self-efficacy Scale. J. Career Assess..

[26] Lee E.J., Song Y.S. (2018). Structural relationship among self-leadership, social support and school adjustment impacting on academic achievement of university students-focusing on the case of S university. J. Vocat. Educ. Res..

[27] Park S.Y., Kim J.W. (2021). An analysis of structural relationships among college students’ job-seeking stress, career decision-making self-efficacy, adjustment to college life, depression, and suicidal ideation. Korea J. Youth Counsel..

[28] Lee M.Y. (2019). The effects of stress on smartphone addiction in university students-mediator effects of depression. J. Korea Contents Assoc..

[29] Seo H.J., Lee H.Y. (2021). The relationships among grit, career barriers, and career preparation behavior perceived by college students. J. Learn. Cent. Curr. Instr..

[30] Kim S.H., Son C.N. (2018). Mediating effects of psychological flexibility and entrapment on the relationship between college students’ life stress and adjustment to college life. J. Digi. Converg..

[31] Ngai S.S., Wang L., Cheung C.K., Mo J., Ng Y.H., Wang P. (2021). Development and validation of the youth career development competency scale: A study based on Hong Kong youth. Int. J. Environ. Res. Public Health..

[32] Árbol J.R., Ruiz-Osta A., Montoro Aguilar C.I. (2021). Personality traits, cognitive styles, coping strategies, and psychological impact of the COVID-19 pandemic lockdown on healthy youngsters. Behav. Sci..

[33] Wang X., Gray M.A., Kim M., Lee S. (2021). Simplifying the measurement of college students’ career planning: The development of Career Student Planning Scale during the COVID-19 pandemic. Exp. Results.

